# Real-Time Estimation of Pathological Tremor Parameters from Gyroscope Data

**DOI:** 10.3390/s100302129

**Published:** 2010-03-16

**Authors:** Juan A. Gallego, Eduardo Rocon, Javier O. Roa, Juan C. Moreno, José L. Pons

**Affiliations:** Bioengineering Group, Consejo Superior de Investigaciones Científicas, CSIC, Ctra. Campo Real, km 0.2 La Poveda, 28500, Arganda del Rey, Spain; E-Mails: erocon@iai.csic.es (E.R.); javieroa@iai.csic.es (J.O.R.); moreno@iai.csic.es (J.C.M.); jlpons@iai.csic.es (J.L.P.)

**Keywords:** tremor, inertial sensors, MEMS gyroscope, tremor modelling, voluntary movement estimation, adaptive signal processing, Kalman filter, real-time estimation, neuroprosthesis

## Abstract

This paper presents a two stage algorithm for real-time estimation of instantaneous tremor parameters from gyroscope recordings. Gyroscopes possess the advantage of providing directly joint rotational speed, overcoming the limitations of traditional tremor recording based on accelerometers. The proposed algorithm first extracts tremor patterns from raw angular data, and afterwards estimates its instantaneous amplitude and frequency. Real-time separation of voluntary and tremorous motion relies on their different frequency contents, whereas tremor modelling is based on an adaptive LMS algorithm and a Kalman filter. Tremor parameters will be employed to drive a neuroprosthesis for tremor suppression based on biomechanical loading.

## 1. Introduction

Tremor is defined as a rhythmic oscillation of a body part [1]. Despite we all have a small component of tremor, the so-called physiological tremor, there exist pathologies with very disabling forms of tremor. Pathological tremor constitutes the most common movement disorder and is continuously increasing its prevalence with ageing. Although not life threatening, tremor often causes social embarrassment and functional disability; in fact, 65% of patients suffering from upper limb tremor report serious difficulties in performing their activities of daily living [2].

Regarding its aetiology, tremors are caused by the different combinations of four different mechanisms: (1) mechanically induced oscillations, (2) oscillations due to reflexes, (3) oscillations due to central neuronal circuits, and (4) oscillations because of disturbed feedback or feedforward loops [1]. However, understanding of the origins of tremors is far from complete.

Pathological tremors are typically classified by position/motor behavior, which reflects under which circumstances tremor appears. According to this criterium, tremor falls into three categories: rest, postural and kinetic tremor [3].

Current strategies on the treatment of tremors are based on drugs, surgery (thalatomy), or deep brain stimulation. The last two options are typically employed in patients refractory to drugs [4]. However, (1) tremor is not managed satisfactorily in 25 % of patients, (2) drugs used often induce side effects, and (3) neurosurgery is associated with hemorrhages and psychiatric manifestations, such as an increased suicidal tendency [5]. These reasons make research on new therapeutic options to manage tremor mandatory.

In this context, functional compensation of pathological tremors via biomechanical loading has appeared as a promising alternative. It is based on the fact that most types of tremors change their characteristics (amplitude and frequency) when the apparent limb impedance is modified, for example, applying a force or adding a mass [6–8]. In particular, it has been clinically demonstrated that increase of damping and/or inertia in the upper limb leads to an effective tremor reduction [2,8]. In [9], the authors have validated, both functionally and clinically, the concept of tremor suppression by means of a wearable robot that applies biomechanical loads in the upper limb. To achieve satisfactory tremor suppression, the wearable robot must leave concomitant voluntary movement unmodified, compensating for tremor. In this regard, real-time estimation of instantaneous tremor parameters becomes fundamental to develop robust orthotic or neuroprosthetic solutions for tremor suppression. The present work is carried out in the framework of EU project TREMOR (ICT–2007–224051), which aims at developing a soft wearable robot for tremor suppression based on a textile substrate. Tremor suppression will be achieved through the application of selective biomechanical loads by means of Functional Electrical Stimulation (FES). FES constitutes a means of activating motoneurons or reflex pathways by stimulating sensory nerve fibers [10]; it therefore allows for employing human muscles as actuators for a wearable robot [11]. The algorithm presented here will be employed to derive tremor characteristics, in order to generate adequate stimulation patterns that counteract tremor, leaving concomitant voluntary movement unaffected.

Inertial sensors technologies constitute a breakthrough and optimal approach to worn sensors in motion analysis [12], and wearable robotics [13]. Advances in microelectromechanical systems (MEMS) permitted to develop miniaturized low cost sensors that measure changes in velocity, position and accelration [13]. Low size and weight allow for wearability, overcoming the limitation of traditional motion capture systems, based on optic sensors and markers, to work in reduced areas. The most spread types of MEMS sensors are solid state accelerometers, gyroscopes, and magnetometers. MEMS accelerometers, and most recently gyroscopes, have been employed to analyze tremorous movements.

Other solutions for kinematic recording of tremors found in the literature are instrumented, ad-hoc devices, such as the instrumented glove [14], or digitizing tablets [15]. Works that study tremor by generating it with an actuated mechanical setup, employ specific sensors technologies such as Hall effect transducers [16], or piezoelectric accelerometers [17], to measure it.

Regarding tremor monitoring with MEMS inertial sensors, accelerometers constitute the most spread alternative [18,19], although it is not the most adequate, [20]. In fact, accelerometers measure limb orientation with respect to gravity, but their measurement is corrupted by voluntary movements, decreasing accuracy dramatically. Moreover, accelerometers measure linear acceleration, whereas articular motions are rotational about joints. Accelerometer data is typically considered to be compound of three factors: linear acceleration, gravity, and white additive noise [21]. No analytic model to distinguish between accelerometer data due to acceleration and gravity is available, although the most common approach is to model acceleration changes as a low pass filter [21,22], or to separate both components by means of a FIR low pass filter [23]. On the contrary, solid state gyroscopes provide a direct measurement of a rotational movement, uninfluenced by gravity [24], but affected by a characteristic low frequency bias. This bias is traditionally thought of being caused by the effects of temperature and mechanical wear [21], although our experiments demonstrate that it is only correlated with temperature, as in next Section.

Most of currently existing tremor estimation algorithms have been developed for cancelling physiological tremor in human-machine interfaces. Physiological tremor has another aetiology than pathological tremors, and possesses also different characteristics: it is barely visible to the unaided eye, and has frequency between 8 and 12 Hz [25]. Physiological tremor is only symptomatic during high precision tasks, thus it is typically employed during hand held surgery. According to this, successful algorithms for cancelling physiological tremors such as the classical Weighted frequency Fourier Linear Combiner (WFLC) [16], Bandlimited Multiple Fourier Linear Combiner (BMFLC) [17], or Double adaptive BM-FLC [26], may not constitute the optimal solution for estimation of pathological tremor parameters. In fact, in [9], Rocon and colleagues employ a preliminary filtering stage that eliminated volitional motion from the input signal before feeding it into a WFLC, which was employed to track pathological tremor. A similar approach has been recently described in [27].

In this paper, we present a two stage algorithm that estimates instantaneous tremor parameters from wearable gyroscope data. As above mentioned, information about current tremor amplitude and frequency will be employed to drive a neuroprosthesis for tremor suppression based on application of selective biomechanical loads. We employ MEMS gyroscopes to record joint kinematics. Next, tremorous patterns are extracted from the recorded motion, based on the frequency distribution of voluntary and tremorous components of movement. The second stage of the algorithm estimates tremor amplitude and frequency from the tremorous component of joint motion. Robust, precise and low delay estimation of tremor parameters is achieved: average amplitude estimation error is 0.001 ± 0.002 rad/s, and frequency estimation agrees with spectrograms.

This paper is organized as follows. First, Section 2 presents selection of sensors, patients, and clinical and functional tests to be recorded. Next, in Section 3, we introduce the architecture of our two stage algorithm, and a series of estimators that will be evaluated for voluntary movement estimation and tremor modelling (each of the stages). The performance of these algorithms when tracking tremor parameters in real data is compared in Section 4; which serves to define an optimal architecture for the algorithm. Finally, we end the paper with a discussion on the results, and some conclusions summarizing major achievements.

## 2. Methods

In order to assess time varying tremor parameters, we record hand motion by measuring wrist flexion-extension. We decide to measure wrist tremor because: (1) tremors are more explicit at distal joints [1], (2) wrist tremor has the largest impact on disability [28], and (3) it constitutes, together with finger tremor, the most studied tremor in clinical literature.

**Figure 1.**
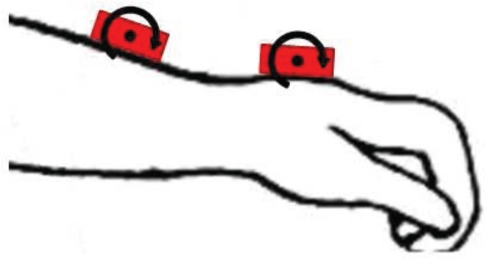
Placement of MEMS gyroscopes (red boxes) for recording wrist flexion-extension. A differential measurement directly provides wrist rotation.

Wrist rotation is obtained with two solid state gyroscopes, placed on the distal part of the forearm, and on the hand, Figure 1. Fixation on soft tissues is avoided in order to eliminate the undesired oscillations they create, and their intrinsic low pass filtering behavior [29]. Sensors are fastened with adhesive tape.

**Figure 2.**
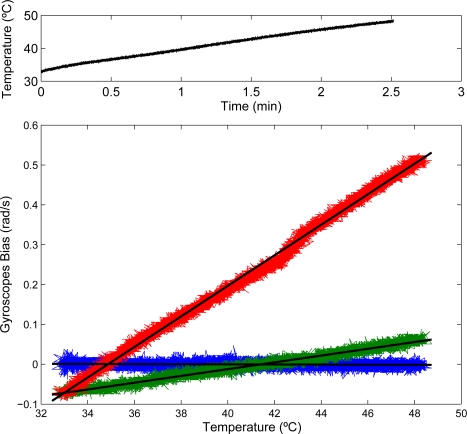
Relationship between gyroscope offset and temperature. Top plot shows temperature variation measured by the IMU. Bottom plot shows correlation between offset and temperature for X (blue), Y (green) and Z (red) axis gyroscopes. High correlation is observed.

Proper alignment between gyroscope axes and wrist joint is ensured before recording. Wrist flexion-extension is simply calculated subtracting the rotation measured with the hand gyroscope from the forearm rotation. Gyroscope bias is compensated online, based on its correlation with temperature, which is measured by the sensor itself. Correlation between gyroscope bias and temperature in three axes of one of the inertial measurement units (IMUs) we employ is shown in Figure 2. Regression coefficients for the three gyroscopes in one of the chipsets are given in Equations 1 to 3, where *b_axis_* represents the bias for each axis, and *T* temperature in °C.

(1) $$b_{x}=-0.000154\cdot T+\;0.00562$$

(2) $$b_{y}=0.008506\cdot T-0.35245$$

(3) $$b_{z}=0.038312\cdot T-0.13358$$

Sensors selected are off the shelf inertial measurement units (IMU) provided by Technaid S.L. They are compound of triaxial accelerometers, gyroscopes, and magnetometers. The low weight of these IMUs, around 40 g, makes them an optimal solution for our application, as tremor would change its characteristics if a larger mass were attached to the limbs [7]. Moreover, their small size (27 mm) does not interfere with user’s movements. As above mentioned, only gyroscope recordings is considered. IMU sensors communicate with a hub trough Controller Area Network (CAN) bus. The host PC receives data from the hub through a USB interface at a sampling frequency of 1 kHz.

### 2.1. Selection of Patients

A group of patients affected by the most common pathologies that cause tremor was recruited for this study. The patients group was compound of four men and one woman, ages ranging from 48 to 74 years old (average 63 years old). All of them agreed to participate in the experiments, and gave their written informed consent. Patients had been previously diagnosed by the neurological personnel of the Hôpital Erasme at Brussels; their clinical features are depicted in Table 1. Patients kept their regular medications, to avoid tremor fluctuations related to changes in therapies. Tremor severity was assessed according to Faher scale [30]. The Ethical Committee of Hôptial Erasme gave ethical approval for this study.

### 2.2. Clinical and Functional Tasks

We selected four tasks that are relevant either from the clinical or usability analysis standpoint. Three of them are employed by neurologists to activate the different types of tremor [19], whereas the fourth one serves to evaluate patients’ ability to perform their activities of daily living [31].

During the whole session, patients were sitting, with both arms comfortably resting on their lap. Selected tasks were:

1. Arms outstretched: The patient is asked to elevate both arms and hold them against gravity with fingers abducted, hands in supination, during 30 s. This task is typically employed to activate postural tremor.
2. Finger to nose: The patient is asked to alternatively touch his nose and knee with the tip of his/her finger during 30 s. The patient must keep contact with nose and knee during a few seconds. This task is typically used to activate kinetic tremor.
3. Rest: The patient is asked to keep both arms resting on the lap during 30 s. The elbow is flexed around 90°. This tasks is typically used to activate rest tremor.
4. Pouring water into a glass: The patient is asked to pour 20 cl water from a standard bottle into a regular glass. The patient could choose how to perform the task, *i.e.*, how to hold the bottle and the glass. This task is selected for functional and usability analysis.

## 3. Real-Time Estimation of Instantaneous Tremor Parameters

This section presents our two stage algorithm for real-time modelling of tremor. It relies on two assumptions: (1) pathological tremors and voluntary motions have different frequency distributions, and (2) pathological tremor constitutes, from a signal processing standpoint, additive noise superimposed to volitional movement [32].

**Figure 3.**
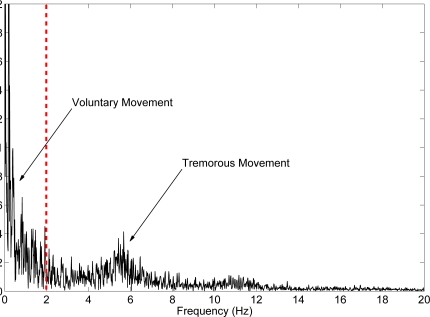
Power spectral density of wrist rotation during a finger to nose task performed by patient 01. It is observed that voluntary movement (below 2 Hz) has considerably more energy than tremorous movement (centered around 5.5 Hz). Dashed red line separates energy attributed to voluntary (left) and tremorous motion (right).

The data we collected yields that pathological tremors occur in a frequency band higher than voluntary motion, Figure 3, which is in agreement with the literature. For example, one large study carried out with young and elderly healthy people and patients suffering from tremor, demonstrates that both groups are able to perform tracking tasks with a frequency up to 2 Hz, the bandwidth decreasing with age [33]. Also in [34], the authors perform a large spectral analysis of twenty four activities of daily living, showing that most of them involve wrist notion in a frequency range around 1 Hz, being the predominant frequency components between 0.48 and 2.47 Hz.

In addition, pathological tremors are reported to occur at higher frequencies, typically in the 3–12 Hz band [1,19]. Moreover, there exists a relationship between the underlying pathology and tremor frequency. For example, Parkinsonian tremor frequency lies within 4–7 Hz, cerebellar tremors manifest between 4 and 6 Hz, and essential tremors broaden to basically the whole 3–12 Hz band [1].

**Figure 4.**
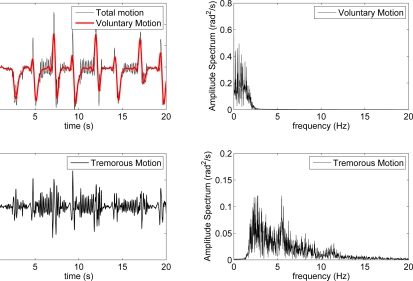
Separation of voluntary and tremorous components of movement by means of recursive digital filters. Top left figure shows the original signal (black) and voluntary movement (red) obtained with a zero phase low pass filter, *f_c_* = 2 Hz. Bottom left plot shows tremorous movement obtained by subtracting voluntary movement from the original signal. Right plots show power spectral densities of voluntary (top) and tremorous components (bottom).

According to this, it is possible to extract voluntary and/or tremorous motion from kinematic data time series based solely on their different frequency contents, for example with a forth and back recursive digital filter that removes one of them from the original signal without causing phase distortion, *i.e.*, delay, Figure 4. Although this approach is not real-time implementable, it sets the basis for our two stage algorithm for estimation of tremor parameters described in next section.

**Figure 5.**
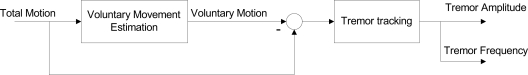
Block diagram of the two stage algorithm for real-time estimation of tremor parameters. First, we generate an estimation of the voluntary component of motion, which subtracted from the total movement yields an estimate of tremor. Afterwards, in stage two, and adaptive algorithm tracks instantaneous tremor parameters.

However, at the moment of developing a real-time algorithm, we must take into account that power of tremorous component of motion is considerably smaller than that of volitional origin. This makes estimation algorithms based on gradient like approaches tend to converge towards the voluntary component, making them unsuitable for direct tremor modelling [33]. Therefore, we need to first isolate the tremorous component of motion. This constitutes the idea of our two stage algorithm: to first generate an estimation of tremorous motion (after stage 1), and next feed this signal into an adaptive algorithm that provides instantaneous tremor frequency and amplitude (stage 2), Figure 5. To generate an estimation of tremor, we employ a tracking algorithm to estimate the voluntary component of motion. Next, based on the fact that tremor alters volitional motion in an additive manner [32], we remove the estimated voluntary movement from the input signal, obtaining an estimation of tremor.

This section reviews a series of algorithms that are employed to develop the two stage algorithm. First, we present a number of techniques to track voluntary movement based on its lower frequency content. Next, we introduce two adaptive algorithms that track an input signal based on Fourier modelling and the Least Mean Square (LMS) recursion, a gradient-like approach [35], and a Kalman Filter that estimates pathological tremor amplitude.

### 3.1. Stage 1. Voluntary Motion Tracking

Two types of tracking algorithms for real-time estimation of voluntary movement will be evaluated. As discussed above, voluntary movements are assumed to be performed between 0 and 2 Hz. Therefore, the tracking algorithm must be designed to neglect any component of the movement over 2 Hz.

Voluntary movement is modelled as a first order process. If we consider a Taylor series that represents voluntary movement, we can neglect the second derivative *ẍ* if either the sampling period *T_s_* or the acceleration itself are small, Equation 4. In our case both assumptions are satisfied: the sampling period is 1 ms, and the maximum of *ẍ* is 2.54 · 10^−4^ rad·s^−3^, 4 orders of magnitude smaller than average angular velocity.

(4) $$x(t)=x\left(t_{n}\right)+T_{s}\dot{x}\left(t_{n}\right)+\frac{T_{s}^{2}}{2}\ddot{x}\left(t_{n}\right)+\cdots$$

#### g–h Filters

g–h filters are simple recursive filters that estimate future position and velocity of a variable based on first order model of the process. Measurements are used to correct these predictions, minimizing the estimation error. Traditional applications of g–h filters are radar tracking and aeronautics [36]. The general form of a g–h filter is:

(5) $$x_{k,k}=x_{k,k-1}+g_{k}\left(y_{k}-x_{k,k-1}\right)$$

(6) $${\dot{x}}_{k,k}={\dot{x}}_{k,k-1}+\frac{h_{k}}{T_{s}}\left(y_{k}-x_{k,k-1}\right)$$

(7) $$x_{k+1,k}=x_{k,k}+T_{s}{\dot{x}}_{k,k}$$

(8) $${\dot{x}}_{k+1,k}={\dot{x}}_{k,k}$$

Equations 5 and 6 are designated as update, tracking, or filtering equations. They estimate the current position and velocity of the variable, *x_k,k_*, *ẋ_k,k_*, based on previous predicted position and velocity, *x*_*k,k*−1_, *ẋ*_*k,k*−1_, taking current measurement *y_k_* into account. Confidence on measures is weighted by gains *g_k_* and *h_k_*. Equations 7 and 8 are called prediction equations because they provide a prediction of future position and velocity, *x*_*k*+1,*k*_, *ẋ*_*k*+1,*k*_, based on first order dynamic model of the variable. As g–h trackers consider a constant velocity model, predicted velocity *ẋ*_*k*+1,*k*_ is equal to the current one, *ẋ_k,k_*.

g–h filters are affected by two error sources [36]: (1) the lag, dynamic, bias or systematic error, which is related to the constant velocity assumption, and (2) the measurement error, which is inherent to the sensor and measurement process. Typically, the smaller *g_k_* and *h_k_* are, the larger is the dynamic error and the smaller are the measurement errors [36]. Therefore, in designing a g–h tracking filter there is a degree of freedom in choice of the relative magnitude of the measurement and dynamic errors. To simplify the selection of gains, we consider two filters that are optimal in some sense. These filters are the Benedict–Bordner Filter and the Critically Dampened filter, described next.

- Benedict–Bordner Filter: The Benedict–Bordner Filter (BBF) minimizes the total transient error, defined as the weighted sum of the total transient error and the variance of prediction error due to measurement noise errors [37]. The BBF is the constant g–h filter that satisfies:
  (9) $$h=\frac{g^{2}}{2-g}$$
  As Equation 9 relates *g* and *h*, the BBF has one degree of freedom. Because for g–h filters increasing the value of *g* diminishes the transient error, the larger *g*, the higher frequencies the BBF tracks.
- Critically Dampened Filter: The Critically Dampened Filter (CDF) minimizes the least squares fitting line of previous measurements [36], giving old data lesser significance when forming the total error sum. This is achieved with weight factor *θ*. Parameters in the g–h filter are related by:
  (10) $$\begin{array}{l} g=1-\theta^{2}\\ h={\left(1-\theta \right)}^{2} \end{array}$$
  Selection of filter gain for the CDF is analogous to that for the BBF.

#### Kalman Filter

The Kalman filter (KF) is the most widespread estimation algorithm, and is employed in a large number of applications. We implement a KF that tracks voluntary movement modelled as a first order process, Equation 4. Therefore, state vector x(*t*) is composed by the variable to be estimated, and its derivative. The problem is formulated as:

(11) $${\hat{x}}_{k,k-1}=\left[\begin{array}{ll} 1 & T_{s}\\ 0 & 1 \end{array}\right]\;{\hat{x}}_{k-1,k-1}$$

(12) $$y_{k}=\left[\begin{array}{cc} 1 & 0 \end{array}\right]\;{\hat{x}}_{k-1,k-1}$$

Covariance matrices are defined taking into account the following considerations:

1. Measurement noise covariance **R**(*k*): as voluntary motion is the variable we are tracking, tremor is assumed to be sensor noise. The value of the measurement noise covariance is considered to be the average covariance of isolated tremor data; therefore $R(k)=\sigma_{\omega }^{2}=0.0643\;{\text{rad}}^{2}\cdot s^{-2}$.
2. Process noise covariance **Q**(*k*): we hypothesize that process noise is related to voluntary motion changes due to tremor. A piecewise constant acceleration model is considered, [38]. This model assumes that voluntary movement undergoes constant and uncorrelated acceleration changes between samples in the form of:
  (13) $$Q=\sigma_{\nu }^{2}\left[\begin{array}{cc} \frac{T_{s}^{4}}{4} & \frac{T_{s}^{3}}{2}\\ \frac{T_{s}^{3}}{2} & T_{s}^{2} \end{array}\right]$$
  To select the variance of the random velocity component, $\sigma_{\nu }^{2}$, we follow the recommendation in [38]: 0.5 · max*_ẍ_* ≤ |*σ_ν_*| ≤ max*_ẍ_*. The second derivative of the raw recorded motion yields that max*_ẍ_* = 0.1042 rad·s^−3^.

### 3.2. Stage 2. Tremor Modelling

State of the art tremor modelling algorithms rely on a time-varying Fourier series, which parameters are estimated recursively. Adaptation to the input signal is based on the LMS algorithm developed by Widrow [35]. As the LMS technique is a descend method that relies on a special estimate of the gradient [35], high energy voluntary motion must be removed first, to ensure proper tremor tracking. The first part of the two stage algorithm accomplishes this task, Figure 5.

We evaluate the performance of two algorithms originally developed to track physiological tremor, and of a Kalman Filter we have developed to estimate tremor amplitude, the parameter that is more prone to change during the execution of a task, while tremor frequency keeps within a ±1.5 Hz interval around tremor frequency [39].

#### Weighted Frequency Fourier Linear Combiner

The Weighted Frequency Fourier Linear Combiner (WFLC) is the most widespread algorithm for tremor modelling. It consists in an extension of the classical noise canceller presented in [35], the Fourier Linear Combiner [40], which also tracks frequency of the input signal based on a LMS recursion. Therefore, the WFLC adapts in real-time its amplitude, frequency and phase [16]:

(14) $$x_{r_{k}}=\{\begin{array}{cc} \text{sin}\;\left(r\;\sum_{t=1}^{k}\omega_{0_{t}}\right), & 1\leq r\leq M\\ \text{cos}\;\left(r\;\sum_{t=1}^{k}\omega_{0_{t}}\right), & M+1\leq r\leq 2M \end{array}$$

(15) $${ɛ}_{k}=s_{k}-W_{k}^{T}X_{k}-\mu_{b}$$

(16) $$\omega_{0_{k+1}}=\omega_{0_{k}}+2\mu_{0}{ɛ}_{k}\sum_{r=1}^{M}r\;\left(w_{r_{k}}x_{M+r_{k}}-w_{M+r_{k}}\;x_{r_{k}}\right)$$

(17) $$W_{k+1}=W_{k}+2\mu_{1}{ɛ}_{k}X_{k}$$

Equation 14 represents the time varying sinusoidal terms of the Fourier Series. Equation 15 defines the error to be minimized by the LMS recursion. Equations 16 and 17 represent frequency and amplitude adaptation. The WFLC has four parameters: the number of harmonics of the model, *M*, the amplitude and frequency adaptation gains, *μ*_0_, and *μ*_1_, and a bias weight *μ_b_* that is included to compensate for low frequency errors [16,41]. The number of harmonics is typically fixed to 1, the other parameters are selected based on experimental data.

#### Bandlimited Multiple Fourier Linear Combiner

The Bandlimited Multiple Fourier Linear Combiner (BMFLC) is a more recent algorithm derived from the FLC. It emerged to compensate for the limitations of the WFLC to track physiological tremor when two constituent frequencies [25] are clearly evident, or when frequency variations occur abruptly [17]. The BMFLC consists in a bank of FLCs that track the input signal based on multiple frequency components. Therefore, each FLC adapts its amplitude to the input signal, although its frequency remains constant.

The performance of the BMFLC relies on the multiple fixed frequencies it can track. An interval is thus defined with the lower and upper frequency of the FLCs bank, *ω*_0_, and *ω_f_*. The number of FLCs in between is defined by parameter *G*. The BMFLC is formulated as follows [17]:

(18) $$x_{r_{k}}=\{\begin{array}{cc} \text{sin}\;\left(\omega_{0}+\left(\omega_{f}-\omega_{0}\right)\frac{r-1}{G+1}\;k\right), & 1\leq r\leq M\\ \text{cos}\;\left(\omega_{0}+\left(\omega_{f}-\omega_{0}\right)\frac{r-1}{G+1}\;k\right), & M+1\leq r\leq 2M \end{array}$$

(19) $${ɛ}_{k}=s_{k}-W_{k}^{T}X_{k}-\mu_{b}$$

(20) $$W_{k+1}=W_{k}+2\mu {ɛ}_{k}X_{k}$$

Equation 18 represents the sinusoidal terms of the Fourier Series. Equation 19 defines the error to be minimized by the LMS recursion. Equation 20 represents amplitude adaptation. The BMFLC has six parameters: the number of harmonics of each FLC model, *M*, amplitude adaptation gain, *μ*, the lower and upper frequency of the FLC bank, *ω*_0_, and *ω_f_*, and the number of filters in between, *G*. A bias weight *μ_b_* is also included to compensate for low frequency errors [16].

Although the BMFLC is not conceived as a frequency tracking algorithm, we developed an equation to estimate the current frequency of the input signal, Equation 21. Frequency estimation is obtained by weighting the contribution of each FLC to amplitude adaptation. For a first order Fourier series, it is expressed as:

(21) $$\omega_{k}=\sum_{r=0}^{L}\frac{\left(a_{r}^{2}+b_{r}^{2}\right)\;\omega_{r}}{\sum_{r=0}^{L}\left(a_{r}^{2}+b_{r}^{2}\right)}$$

#### Kalman Filter

The WFLC and BMFLC algorithms provide adaptation to the input signal based on special estimates of the gradient. On the contrary, Kalman Filter (KF) constitutes the optimal solution for estimation problems, in the sense it minimizes the covariance of a posteriori estimation error. Therefore the performance of WFLC and BMFLC, overall in terms of amplitude estimation, as it is the parameter that varies the most, can be enhanced using an adequate KF. In a similar manner to WFLC, we define our KF as:

(22) $$\left[\begin{array}{c} \omega_{0,k,k-1}\\ A_{k,k-1}\\ B_{k,k-1}\\ {\mathrm{tr}}_{k,k-1} \end{array}\right]=\left[\begin{array}{cccc} 1 & 0 & 0 & 0\\ 0 & 1 & 0 & 0\\ 0 & 0 & 1 & 0\\ 0 & \text{cos}\left(\omega_{0,k,k-1}\right) & \text{sin}\left(\omega_{0,k,k-1}\right) & 0 \end{array}\right]\;\left[\begin{array}{c} \omega_{0,k-1,k-1}\\ A_{k-1,k-1}\\ B_{k-1,k-1}\\ {\mathrm{tr}}_{k-1,k-1} \end{array}\right]$$

(23) $$y_{k}=\left[\begin{array}{cccc} 0 & 0 & 0 & 1 \end{array}\right]\;{\hat{x}}_{k-1,k-1}$$

where *A* and *B* represent the amplitude of the sinusoidal terms of a first order Fourier series, and *ω*_0_ the current tremor frequency. As tremor frequency suffers slow changes, and varies in a ±1.5 Hz interval around a characteristic frequency that depends on the patient, [39], WFLC frequency estimation is employed. Therefore we define a cascade filter that consists on a WFLC that tracks tremor frequency, and feds it into the Kalman Filter that estimates tremor amplitude.

Covariance matrices are adjusted as follows:

1. Measurement noise covariance $R(k)=\sigma_{\tau }^{2}$, which has only slight impact on transient duration.
2. Process noise covariance is defined as **Q**(*k*) = diag(*q*_1,1_, *q*_2,2_, *q*_3,3_, *q*_4,4_), because state variables are considered to be mutually independent.

## 4. Results

This section presents evaluation of the algorithms described in previous section for both voluntary motion tracking, and estimation of tremor parameters. The idea is to find a unique filter setup that provides accurate tracking of instantaneous tremor parameters for every patient and task. To do so, first, we present the figure of merit that will be employed to tune each algorithm, and compare the performance of different candidates. Next, we summarize the results obtained with each of them.

### 4.1. Evaluation of Voluntary Movement Tracking Algorithms

The Kinematic Tracking Error (KTE) evaluates the smoothness, response time, and execution time of a tracking algorithm, [41]. It is expressed mathematically as:

(24) $$\text{KTE}=\sqrt{\varphi_{\left|b^{*}\right|}^{2}+\sigma_{\left|b^{*}\right|}^{2}}$$

where *φ*_|b*|_ and $\sigma_{\left|b^{*}\right|}^{2}$ are the mean and variance of the absolute estimation error, b* = |*y_k_* − *x*_*k*+1,*k*_|, respectively. The former measures how fast the algorithm is capable of reacting when the velocity changes, whereas the latter quantifies the smoothness or filtering of the estimated variable [41]. Offline voluntary motion estimation obtained with a forth and back recursive filter is employed as the reference signal the estimators should track. This technique consists in filtering input data in both the forward and reverse directions; after filtering in the forward direction, the algorithm reverses the filtered sequence and runs it back through the filter, which yields precisely zero-phase distortion.

First, we present results obtained with the optimal parameter(s) for each of the voluntary movement estimation algorithms presented in Section 3.1 The condition to select the optimal parameter(s) for each algorithm is to find the tuning that minimizes the KTE between filter estimation of voluntary motion and zero phase (off-line) recursive estimation of voluntary movement. Optimal filters are:

- Benedict–Bordner Filter with *g* = 0.018.
- Critically Dampened Filter with *θ* = 0.990.
- Kalman Filter with $\sigma_{\omega }^{2}=0.0643$ and $\sigma_{\nu }^{2}=0.1042$.

The KTE is employed next to compare their relative performance. Table 2 summarizes KTE per kind of task and patient, for the optimal setup for the three algorithms. We observe that among the BBF, CDF, and KF, the CDF with *θ* = 0.990 performs the best when tracking voluntary movement, as it provides the least KTE for all tasks; thus it is the approach we select for the first stage of the algorithm.

**Figure 6.**
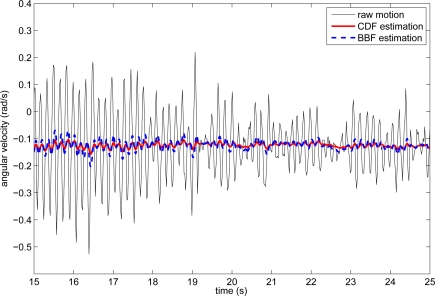
Comparison between CDF and BBF estimation of voluntary movement from raw gyroscope data during an arms outstretched test performed by patient 01.

Figure 6 shows an example of CDF and BBF estimation of voluntary movement from raw gyroscope recording, during an arms outstretched task performed by patient 01. We observe that BBF estimation is less smooth than that of the CDF for a similar adaptation to transitory changes.

### 4.2. Evaluation of Tremor Modelling Algorithms

An optimal figure of merit to evaluate tremor estimation algorithms must consider the physical nature of the estimation error; for example if it originates from phase difference between real and estimated tremor, or estimation overshoots and undershoots. Traditional use of root mean square error (RMSE) suffers from these problems: (1) because errors due to undershoots and overshoots posses large power, which make them overshadow errors from interest, and (2) because the presence of delays affects the RMSE severely, although it does not necessarily indicate poor performance [42]. In this regard, the so-called filtered mean square error with delay correction, FMSE_d_, takes both phenomena into account [42], as it first aligns estimated tremor with the reference signal, and afterwards computes the delay corrected estimation error. The FMSE_d_ is defined as follows:

(25) $${\text{FMSE}}_{d}=\sqrt{{\left(s_{k}-t_{k-{\hat{d}}_{k}}\right)}^{2}}$$

where *s_k_* represents the input tremor signal to be estimated, and *t*_*k−d̂_k_*_ stands for the delay compensated tremor estimation. Instantaneous delay *d̂_k_* is calculated offline by means of an adaptive algorithm that minimizes the mean square error function based on a LMS-like recursion, [43]. As mentioned before, once instantaneous delay is obtained, RMSE between delay corrected estimation of tremor and the reference signal obtained is computed, providing the FMSE_d_.

First we select filter parameters so that they minimize FMSE_d_, and afterwards we compare the performance of each filter presented in Section 3.2 Optimal filters are:

- Weighted Frequency Fourier Linear Combiner with *μ*_0_ = 5 · 10^−4^, *μ*_1_ = 2 · 10^−2^, *μ_b_* = 1 · 10^−2^, *M* = 1, *f*_0_ = 6 Hz.
- Bandlimited Multiple Fourier Linear Combiner with *μ* = 4 · 10^−2^, *μ_b_* = 0, *M* = 1, *f*_0_ = 3 Hz, *f_n_* = 8 Hz, *G* = 4.
- Kalman Filter with *μ*_0_ = 5 · 10^−4^, *μ*_1_ = 1 · 10^−2^, *μ_b_* = 1 · 10^−2^, *M* = 1, *f*_0_ = 6 Hz, **R** = 0.01, **Q** = (1, 1, 1, 1).

Table 3 summarizes FMSE_d_ per task and patient for the optimal setup for the three algorithms. FMSE_d_ is computed as the error between applying the different tremor modelling algorithms to the estimated tremor, obtained as the difference between raw gyroscope signal and CDF estimation, and the zero phase offline estimation of tremor. We observe that among the WFLC, BMFLC, and KF, the KF (with a previous WFLC stage) performs the best when estimating instantaneous tremor amplitude, as it provides the least FMSE_d_ for all tasks, and also is capable of tracking tremor frequency in a robust fashion, as shown below. Therefore, we choose to employ a WFLC in cascade with a KF to estimate tremor parameters in real-time.

**Figure 7.**
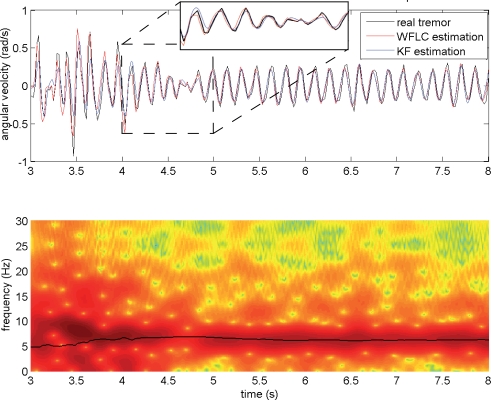
Top plot: Comparison between tremor amplitude tracking with the WFLC (blue line), and a cascade algorithm compound by a KF preceded by a WFLC (red line). Bottom plot: frequency tracking with the WFLC (solid line), plotted against tremor spectrogram. Data corresponds to an arms outstretched test performed by patient 01.

Figure 7 shows tremor amplitude and frequency estimation obtained with the WFLC alone, and together with the KF, during an arms outstretched task performed by patient 01. We observe that the WFLC is capable of adapting to tremor frequency and reacting when changes occur (bottom plot), and that KF adapts faster than WFLC when tremor amplitude varies. This happens because of the optimal nature of the KF, which makes it adjust its gain continuously, on the contrary to the WFLC that has a fixed gain to descent the gradient.

## 5. Discussion

Previous sections presented a two stage algorithm for real-time estimation of tremor parameters. The first stage is in charge of separating voluntary and tremorous components of movement, based on the fact that tremor alters volitional motion in an additive manner [32]. Next, we generate an estimation of tremor as the difference between raw motion and this estimated voluntary movement. The second stage estimates instantaneous tremor amplitude and frequency from isolated tremorous movement. We proposed and evaluated three algorithms for voluntary movement estimation, and three algorithms for tremor modelling.

Regarding voluntary movement estimation, we evaluated two g–h filters, the Benedict–Bordner (BBF) and Critically Dampened Filters (CDF), and a Kalman Filter (KF), for tracking volitional motion based on a figure of merit that accounts for tracking error and estimation smoothness, the Kinematic Tracking Error (KTE). This analysis yielded that g–h filters outperform the KF, because it results difficult to make it adapt quickly to changing voluntary movement patterns without tracking tremor, which translates into a larger KTE, Table 2. Comparing the performance of CDF and BBF, we observe that CDF outperforms BBF because its intrinsic oscillatory nature, that makes it resonate almost in phase with tremor. This occurs because the CDF has two equally spaced zeroes; thus it behaves as a critically dampened system [36]. This also makes the CDF react faster when changes in volitional movement appear, decreasing estimation error during transitory periods. Therefore, the CDF constitutes the optimal filter during both steady and more dynamically complex tasks, as demonstrated by the fact that it provides the least KTE during both rest and finger to nose tests, Table 2.

Algorithms for estimation of instantaneous tremor frequency and amplitude are evaluated based on the Filtered Mean Square Error with Delay correction (FMSE_d_). This metric presents the advantages of accounting for errors due to undershoots and overshoots, and those originated from estimation delays [42]. First, we evaluated two algorithms originally devoted to physiological tremor tracking, the Weighted Frequency Fourier Linear Combiner (WFLC), and the Bandlimited Multiple Fourier Linear Combiner (BMFLC). Physiological tremor not only has different aetiology than pathological tremors, but also manifests differently in terms of amplitude and frequency [25,26]. Although the WFLC has also been successfully employed in the context of pathological tremor estimation [9], we evaluated a novel approach based on a cascade algorithm compound by a WFLC and a Kalman Filter (KF). This algorithm raised as the best solution in the sense it minimizes FMSE_d_, as it puts together accurate tremor frequency estimation based on a WFLC, and an independent KF for amplitude estimation, Table 3. In fact, FMSE_d_ is at least five time smaller than that obtained with the BMFLC, which constitutes the second best candidate. KF tremor estimation proves to be robust and precise both during steady state regime and transitory periods, while BMFLC and overall WFLC lack of that accurate adaptation during transients, mainly because of the fixed gain they employ. This is demonstrated because performance in terms of FMSE_d_ degrades the most during finger to nose and water glass tests, Table 3.

**Figure 8.**
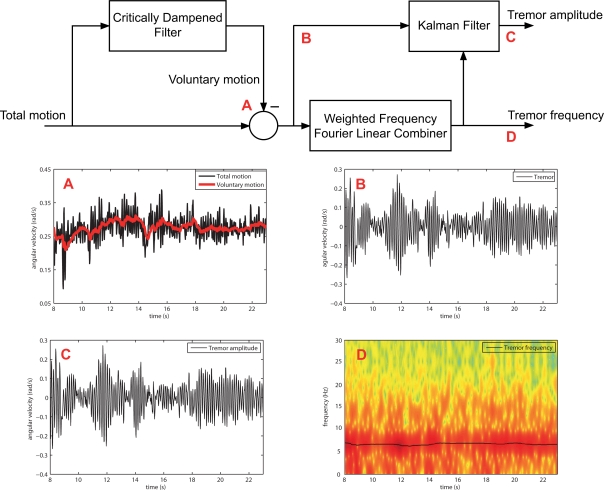
Block diagram summarizing the two stage algorithm for real-time estimation of instantaneous tremor parameters. First, a Critically Dampened Filter estimates voluntary motion from raw kinematic data. Next, we generate an estimation of tremor by subtracting voluntary from raw movement. At the second stage, the Weighted Frequency Fourier Linear Combiner estimates instantaneous tremor frequency, and then feds it into a Kalman Filter that tracks instantaneous tremor amplitude.

Therefore, the optimal architecture for the two-stage algorithm is constituted by a Critically Dampened Filter that estimates voluntary movement, and a cascade algorithm compound by a Weighted Frequency Fourier Linear Combiner that estimates tremor frequency and feds it into a Kalman Filter that tracks tremor amplitude, Figure 8. This approach provides an average tremor estimation error 0.001 ± 0.002 rad/s, with robust frequency tracking if compared to spectrograms.

## 6. Conclusions

This paper presented a two stage algorithm for real-time estimation of instantaneous tremor parameters. At the first stage, the algorithm separates tremorous and concomitant voluntary movement based on their different distributions in the frequency domain. Next, estimated voluntary movement is removed from raw kinematic data, in order to generate an estimation of tremor. This estimation is then fed, at a second stage, into aWeighted Frequency Fourier Linear Combiner (WFLC) that tracks tremor frequency, and into a Kalman Filter that uses WFLC frequency information to estimate tremor amplitude.

The resulting algorithm provides accurate estimation of tremor amplitude, with an average FMSE_d_ of 0.001 ± 0.002 rad/s, and robust frequency estimation when compared with spectrograms. Our two stage algorithm has been validated with patients suffering from major pathologies that cause tremor during the execution of both clinical and functional tasks. Real-time tremor parameters will be employed to drive a “soft robot” or neuroprosthesis for tremor suppression based on Functional Electrical Stimulation.

## Figures and Tables

**Table 1.** Clinical features of enroled patients.

Patient number	Medical History	Tremor	Body segments affected	Frequency	Grade^1^
01	Essential Tremor	Postural	Right upper limb	7 Hz	2
		Kinetic	Right upper limb	4 Hz	2

02	Paraneoplastic Syndrome	Kinetic	Upper/lower limbs	5–6 Hz	1,2
		Postural	Right/left inch	2–3 Hz	1

03	Idiophatic Parkinson	Rest	Upper limbs	3–4 Hz	1,3
		Postural	Left hand	6 Hz	1

04	Extrapyramidal Syndrome	Rest	Upper limbs	3–4 Hz	2
		Postural	Upper limbs	3–4 Hz	1

05	Essential Tremor	Postural	Right upper limb	7 Hz	2
		Kinetic	Upper limbs	4 Hz	2

1 If there are two figures the first one refers to the right limb and the second one to the left limb. If not, tremor severity is the same for both limbs. The grades are: 0: Absent, 1: Discrete and infrequent, not disturbing for the patient, 2: Mild, tremor amplitude is discrete but persistent, or tremor amplitude is mild but its presence is intermittent, 3: Intense, the tremor interferes in some activities, its amplitude is mild but it appears most of the time, 4: Severe, the tremor interferes in most of the chores, its amplitude is high, and it appears most of the time.

**Table 2.** Kinematic Tracking Error (rad/s) for voluntary movement tracking algorithms organized by

Algorithm	Arms Outstretched	Finger to nose	Rest	Water into a glass
Benedict–Bordner Filter	0.194 ± 0.058	0.400 ± 0.134	0.147 ± 0.091	0.291 ± 0.083
Critically Dampened Filter	0.121 ± 0.053	0.372 ± 0.118	0.134 ± 0.081	0.264 ± 0.073
Kalman Filter	0.169 ± 0.100	0.378 ± 0.143	0.174 ± 0.129	0.312 ± 0.124

**Table 3.** Filtered Mean Square Error with Delay correction (rad/s) for tremor estimation algorithms

Algorithm	Arms Outstretched	Finger to nose	Rest	Water into a glass
Weighted Frequency FLC	0.017 ± 0.007	0.052 ± 0.023	0.014 ± 0.006	0.042 ± 0.020
Bandlimited Multiple FLC	0.007 ± 0.008	0.008 ± 0.019	0.005 ± 0.012	0.006 ± 0.013
Kalman Filter	0.001 ± 0.003	0.000 ± 0.002	0.001 ± 0.001	0.001 ± 0.003
